# Investigation of Microstructural Characterization and Tensile Deformation Mechanisms in Inconel 617 Welded Joints Produced by GTAW

**DOI:** 10.3390/ma19061251

**Published:** 2026-03-21

**Authors:** Mingyang Zhao, Lang Wang, Wenhao Ren, Yuxin Wang, Tao Zhang, Zhengzong Chen

**Affiliations:** 1 Research Institute of Special Steels, Central Iron and Steel Research Institute Co., Ltd., Beijing 100081, China; zmy000806@163.com (M.Z.); wenhao_ren@126.com (W.R.); 2 School of Materials and Metallurgy, University of Science and Technology Liaoning, Anshan 114051, China; 3 State Key Laboratory of Advanced Special Steel, Beijing 100081, China; 4 Nuclear Equipment Division, China Nuclear Power Engineering Co., Ltd., Beijing 100840, China; wanglang@cnpe.cc; 5 Lanzhou LS Heavy Equipment Co., Ltd., Lanzhou 730314, China; zhangtao@lshec.com

**Keywords:** Inconel 617 alloy, microstructure, tensile properties, deformation mechanisms

## Abstract

The microstructural evolution and tensile behavior of Inconel 617 welded joints produced by gas tungsten arc welding (GTAW) with ERNiCrCoMo-1 filler were systematically investigated. Detailed microstructural characterization revealed that Cr-rich M_23_C_6_ and Ti-rich MC carbides are the dominant precipitates, while Mo-rich M_6_C forms locally along grain boundaries after thermal exposure. The fusion and weld zones exhibit fine dendritic morphologies with uniformly distributed precipitates, resulting in significant strengthening through precipitation and dislocation–pinning mechanisms. Owing to the low heat input and compositional compatibility between the weld and base metals, the heat-affected zone remains extremely narrow and free of compositional transitions. The welded joint attains tensile strengths of 920 MPa at room temperature and 605.5 MPa at 750 °C, corresponding to joint efficiencies of 117% and 121%, respectively, with fracture consistently occurring in the base metal. Deformation analysis shows that plasticity at room temperature is governed by planar slip and dislocation entanglement, whereas deformation twinning predominates at elevated temperatures owing to the reduced stacking-fault energy and the pinning effect of M_23_C_6_ carbides. These results provide key insights into the deformation and strengthening mechanisms controlling the high-temperature performance of GTAW-welded Inconel 617 joints and offer guidance for their application in advanced nuclear and high-temperature energy systems.

## 1. Introduction

High-temperature alloys play a vital role in the development of nuclear reactors owing to their exceptional strength, oxidation resistance, corrosion resistance, fatigue resistance, creep resistance, and structural stability [1]. Among them, Inconel 617—a Ni-Cr-Co-Mo alloy—is distinguished by its superior high-temperature strength and heat resistance, making it particularly suitable for structural components in chemical processing equipment, nuclear reactors, and aerospace systems [2,3,4,5]. This alloy has been incorporated into the ASME nuclear standards as one of six materials approved for high-temperature nuclear components. Moreover, Inconel 617 is listed in ASME Code Case N-898, which specifies its application in Class A elevated-temperature service under Section III, Division 5, with a design life of 100,000 h. Such comprehensive qualification data further underscores its extensive potential for use in advanced nuclear power systems [6].

With the widespread adoption of Inconel 617 alloy, research on its weldability has attracted increasing attention. Welding is inevitably required at multiple stages during the fabrication of heat exchangers and pipelines for high-temperature gas-cooled reactors [7]. Despite the structural complexity of these components, welding remains indispensable in their manufacture. Considering factors such as joint geometry and component dimensions, the gas tungsten arc welding (GTAW) process has been identified as the most suitable technique. Welded joints play a pivotal role in industrial systems, serving as critical connections between components, and their integrity directly influences overall system performance. Nevertheless, welded joints are well known to exhibit a higher susceptibility to failure under high-temperature conditions, primarily due to microstructural and mechanical heterogeneity across different regions of the joint [8,9].

In recent years, extensive research has been carried out worldwide on the engineering applications of Inconel 617 alloy. Reference [10] reported the welding of 22Cr15Ni3.5CuNbN steel using ERNiCrCoMo-1 filler wire, revealing Ti-rich precipitates in the weld metal and Cr- and Nb-rich precipitates in the base metal. The study further demonstrated that grain refinement induced by welding markedly influences the alloy’s mechanical properties. References [11,12,13,14] examined the mechanical performance and microstructural evolution of dissimilar Inconel 617 welded joints at elevated temperatures. These investigations identified M_23_C_6_ (Cr-rich) and MC (Ti-rich) phases as the dominant precipitates in Inconel 617, while the ERNiCrCoMo-1 filler promotes the formation of Mo-rich M_6_C phases after thermal exposure, thereby significantly modifying the weld microstructure. Softening of the base-metal matrix, resulting from the welding process or deformation-induced precipitate evolution, can cause instability and premature failure of the joint under high-temperature or cyclic loading conditions. Overall, the high alloying content renders the welding of nickel-based superalloys challenging and susceptible to defects. Consequently, systematic evaluation of welding processes and detailed analyses of weld microstructure and mechanical behavior are essential.

In this study, Inconel 617 alloy was welded using the gas tungsten arc welding (GTAW) technique. The precipitates in different regions of the welded joint were characterized both qualitatively and quantitatively. Tensile tests were performed at room temperature and 750 °C, and the fracture morphology and deformation mechanisms of the joints were subsequently examined. By correlating weld joint microstructure, precipitate distribution, and tensile deformation mechanisms with joint efficiency at service-relevant temperatures, this work aims to provide a microstructure-based framework for selecting and qualifying GTAW procedures for Inconel 617 components in advanced nuclear and high-temperature energy systems.

## 2. Materials and Methods

### 2.1. Materials and Experimental Procedures

**Figure 1:**
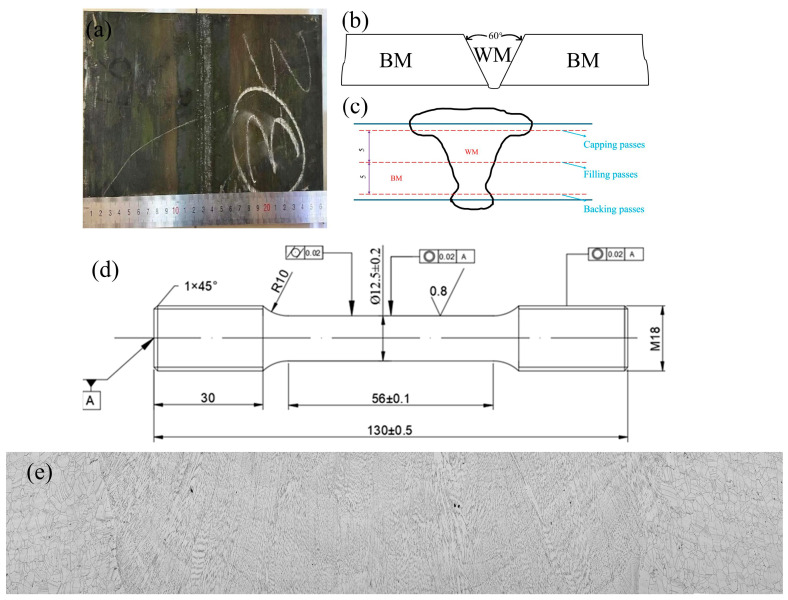
(**a**) Inconel 617 alloy welded joint; (**b**) Schematic diagram of the welding groove; (**c**) Schematic diagram of the microhardness testing; (**d**) Dimensions of the tensile test specimen; (**e**) Inconel 617 alloy welded joint full weld section.

The Inconel 617 alloy ingot was produced through a dual-melting route comprising vacuum induction melting followed by vacuum arc remelting. After subsequent forging and rolling, alloy plates with a thickness of 23 mm were fabricated. Multi-pass, multi-layer welding of these plates was performed using manual GTAW with imported ERNiCrCoMo-1 filler wire (Φ2.4 mm). The welding parameters were as follows: heat input, 0.78 kJ mm^−1^; current, 110–125 A; voltage, 9–13 V; and travel speed, 125–160 mm min^−1^. High-purity argon (99.99%) was used as the shielding gas with a flow rate of 12–15 L·min^−1^, and a 2.4 mm diameter thoriated tungsten electrode (2% ThO_2_) was employed under DCEN polarity. The inter-pass temperature was controlled below 150 °C to ensure consistent microstructural evolution and to minimize residual stresses. The welded plates are shown in Figure 1a. A V-groove configuration with a groove angle of 60° was adopted for the joint, as illustrated in Figure 1b. Post-weld non-destructive testing was conducted in accordance with ASME BPVC Section V [15] and HBB-5000 standards [16]. Plates that passed inspection were subjected to post-weld heat treatment at 980 °C for 3 h. The chemical compositions of the weld metal (WM) and base metal (BM), listed in Table 1, conform to ASME specifications.

Microhardness testing was performed using an FM-300 digital microhardness tester (Kawasaki City, Kanagawa Prefecture, Japan) equipped with the SVDM3 software system. The SVDM3 software system used for data acquisition and analysis with the microhardness tester is provided by FUTURE-TECH Corp. Official product information is available on the manufacturer’s website: https://www.ft-hardness.com/en/index.html (accessed 10 November 2025), in accordance with the GB/T 4340.1 standard [17]. Measurements were taken on the capping, filling, and backing passes of the welded joint, as shown in Figure 1c. The center of the gauge length for all specimens is the weld, and the sides extending to the clamping end are the base material. Room-temperature tensile tests were conducted following ASTM A370-23 [18], while high-temperature tests complied with ASTM E21-20 [19] at a test temperature of 750 °C. All tensile tests were performed at a constant engineering strain rate of $\dot{\varepsilon }$ = 1.0 × 10^−3^ s^−1^. For each testing condition (BM-RT, BM-HT-750, Joint-RT, and Joint-HT-750), at least three tensile specimens (*n* ≥ 3) were tested. The reported tensile properties in Table 2 represent the arithmetic mean values, and the corresponding standard deviations are also provided to reflect the data scatter and repeatability. The geometry of the specimens is shown in Figure 1d.

### 2.2. Material Characterization

The microstructure and precipitates of the Inconel 617 welded joint were characterized using a Leica FEM-4M optical microscope (Leica Microsystems, Wetzlar, Germany) and an FEI Quanta 650 scanning electron microscope (SEM) (FEI Company, Hillsboro, OR, USA), with energy-dispersive X-ray spectroscopy (EDS) employed for phase identification. The morphology and size of the precipitates were further analyzed using Hitachi H-800 (Tokyo, Japan) and F20 transmission electron microscopes (TEM) (FEI Company, Hillsboro, OR, USA), and the precipitate size was measured using Nano measurer software (version 1.2.0.5; accessed via https://nano-measurer.software.informer.com/ on 10 October 2025). Selected area electron diffraction (SAED) was applied to determine the crystal structures of the precipitates. Samples for optical microscopy (OM) and SEM were ground with progressively finer SiC papers, mechanically polished, and etched in a CuCl_2_–HCl–ethanol solution (5 g CuCl_2_ + 100 mL HCl + 100 mL ethanol). TEM foils were prepared by twin-jet electropolishing in a 10% perchloric acid–ethanol solution at −25 °C under a constant current of 80 mA. TEM observations were carried out at an accelerating voltage of 175 kV. After mechanical polishing, the surface of the EBSD specimen is further finished by electrolytic polishing. Electron backscatter diffraction (EBSD) Kikuchi patterns are acquired using a JSM-7200F scanning electron microscope (JEOL Ltd., Tokyo, Japan) equipped with an Oxford NordlysMax2 detector (Oxford Instruments, Abingdon, UK), and the data are analyzed using Aztec software (Oxford Instruments NanoAnalysis, High Wycombe, UK).

## 3. Results and Discussion

### 3.1. Microstructure of the Joint

The relatively low heat input of the gas tungsten arc welding (GTAW) process helps minimize the heat-affected zone (HAZ), particularly during the welding of Inconel 617 alloy [20]. The nominal heat input of 0.78 kJ mm^−1^ used in this work lies at the lower end of the typical range reported for GTAW of Inconel 617 and related Ni-based superalloys, where values between approximately 0.7 and 1.5 kJ mm^−1^ are commonly employed [10]. A relatively low heat input was deliberately selected to promote grain refinement in the weld metal, to suppress excessive HAZ growth and liquation cracking, and to minimize segregation-related defects. Owing to its excellent thermal resistance, high-temperature stability, high melting point, and strong oxidation resistance, Inconel 617 effectively restricts heat diffusion into the base metal during welding. Consequently, the grain structure remains largely unchanged, resulting in a narrow HAZ, as shown in Figure 1e. In addition, the ERNiCrCoMo-1 filler wire exhibits excellent compatibility with Inconel 617, promoting efficient heat transfer between the weld and base metals and further mitigating thermal effects on the microstructure. The precise control inherent to GTAW—together with its relatively slow travel speed and appropriate cooling measures—effectively suppresses HAZ growth. As a result, the HAZ is extremely limited and often indistinct [21,22,23]. Therefore, this study primarily focuses on the microstructure and properties of the weld metal, fusion zone, and base metal.

#### 3.1.1. Microstructure and Analysis of the Base Metal

**Figure 2:**
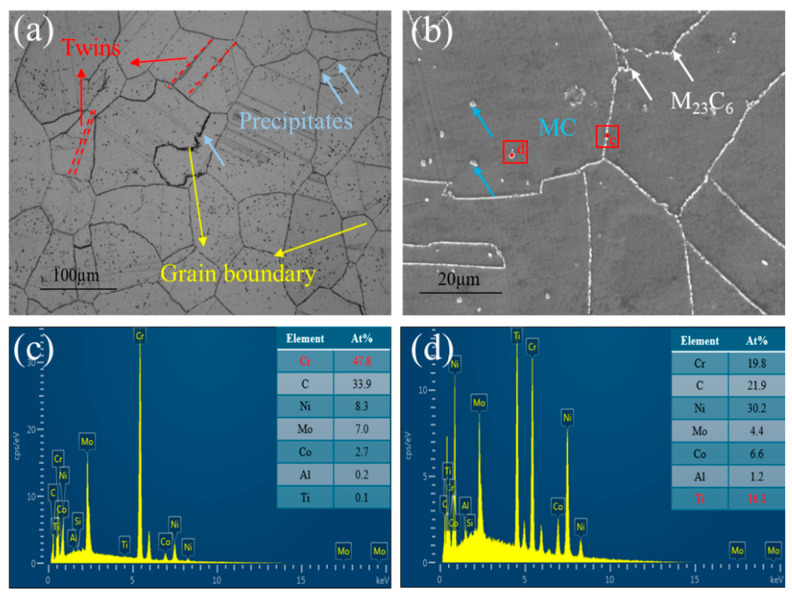
(**a**) Microstructure of the base metal; (**b**) Ti-rich and Cr-rich phases; (**c**) EDS of M_23_C_6_ precipitates; (**d**) EDS of MC precipitates.

Figure 2 presents the microstructure of the Inconel 617 alloy. The optical micrograph (Figure 2a) shows the base metal composed of equiaxed austenitic grains with an average size of approximately 100 μm. Numerous precipitates are distributed both along grain boundaries and within the grains. Many annealing twins are also observed, indicating continued grain growth during post-weld annealing. The presence of twins at grain-growth fronts suggests atomic stacking events associated with recrystallization [24]. The SEM image of the base metal (Figure 2b) reveals similar features. EDS analyses of precipitates at grain boundaries and within grains (Figure 2c and Figure 2d, respectively) show that the grain-boundary precipitates are Cr-rich, whereas the intragranular precipitates are Ti-rich. According to previous studies [21,22], these correspond to M_23_C_6_ and MC carbides, respectively.

**Figure 3:**
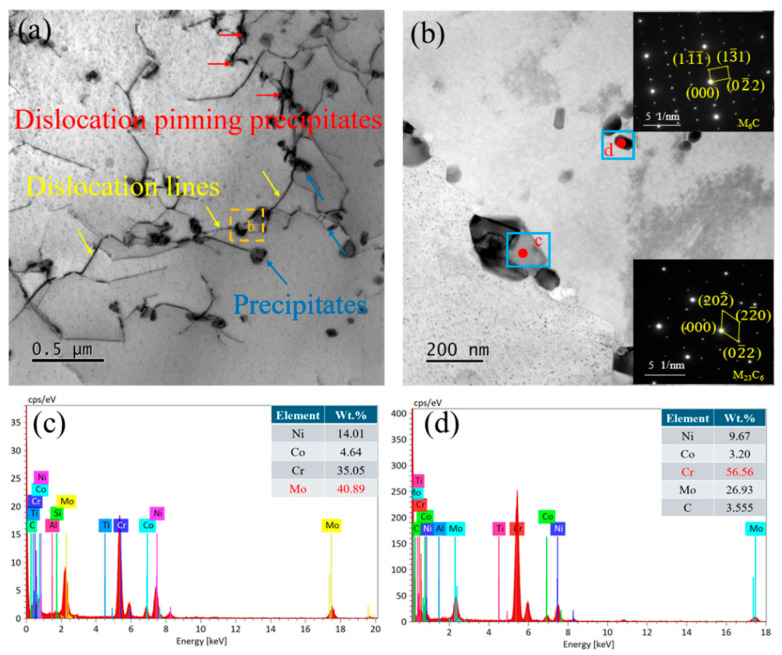
(**a**) TEM image of the base metal; (**b**) TEM images of M_6_C and M_23_C_6_ phases in the base metal; (**c**) EDS of M_6_C precipitates (**d**) EDS of M_23_C_6_ precipitates.

Figure 3 shows the TEM microstructure of the Inconel 617 alloy substrate. In Figure 3a, clear interactions between precipitates and dislocations are observed. The intragranular precipitates exert a pronounced pinning effect on dislocation motion, forming a composite strengthening mechanism based on the “dislocation–precipitate” interaction. Distinct dislocation entanglements are also evident, primarily resulting from intense thermal cycling and stress concentration, which lead to significant local strain accumulation [25]. A higher-magnification image of the selected region (Figure 3b) further characterizes the precipitates distributed along grain boundaries and within grains. SAED and EDS analyses identify the grain-boundary precipitates as Mo-rich M_6_C carbides (Figure 3c), while the intragranular, particle-like precipitates correspond to Cr-rich M_23_C_6_ carbides (Figure 3d), with precipitate sizes of approximately 50~100 nm. The local accumulation of precipitates may induce stress concentration, serving as potential crack initiation sites, particularly under cyclic or sustained loading. Further fracture analysis is required to confirm this behavior.

#### 3.1.2. Microstructure and Analysis of the Fusion Zone

**Figure 4:**
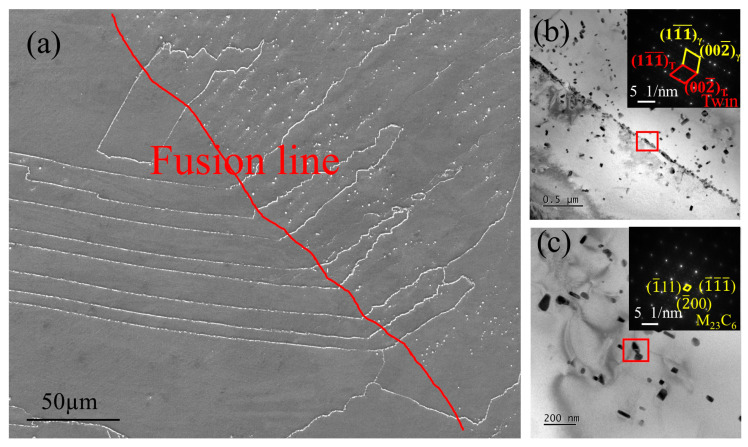
(**a**) SEM image of the weld joint fusion zone; (**b**) TEM image of the twinned structure at the fusion zone; (**c**) TEM image of M_23_C_6_ at the fusion zone.

Figure 4a shows the SEM image of the fusion zone, where a characteristic bright, strip-like structure is observed along the fusion-line direction. This feature is typical of fusion zones and represents the metallurgical bonding region formed by re-melting and solidification between adjacent melt pools during welding. The bright strip structure appears layered, with distinct interlayer boundaries, indicating that during multi-pass and multi-layer deposition, the melt-pool boundaries were not fully eliminated, leading to slight metallurgical discontinuities. As shown in Figure 4b, numerous fine particles are distributed along the fusion line, forming chain-like or lattice arrangements along the interface. The region marked by the red box was analyzed by SAED, revealing a crystalline structure with evident twin characteristics and a regular lattice arrangement. Pronounced grain-boundary deflection in this region suggests that thermal effects near the fusion line induced grain growth or partial recrystallization. The particle precipitates act as dislocation–pinning sites, contributing to local strengthening along the fusion line, although they may also serve as potential sources of brittleness [26]. Figure 4c presents nanoscale precipitates dispersed within the matrix, exhibiting rod-like or particulate morphologies. SAED analysis confirms that these precipitates correspond to the M_23_C_6_ phase, showing specific orientation relationships that indicate preferential growth along certain crystallographic planes during solidification, with precipitate sizes of approximately 50~100 nm. Owing to repeated thermal cycling, the microstructure in the fusion-line region differs significantly from that of the base matrix. The fine precipitates distributed along the fusion line enhance the local strength through precipitation strengthening and dislocation–pinning mechanisms [27].

#### 3.1.3. Microstructure and Analysis of the Weld Metal

**Figure 5:**
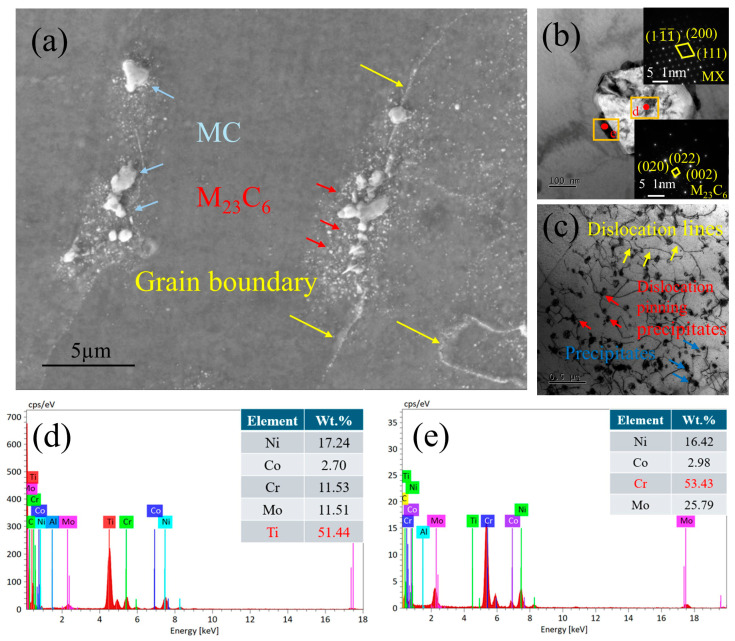
(**a**) SEM image of the weld; (**b**) TEM image of Ti-rich and Cr-rich phases at the weld; (**c**) TEM image showing precipitate pinning dislocations at the weld; (**d**) EDS of MC precipitates; (**e**) EDS of M_23_C_6_ precipitates.

Figure 5 presents the microstructure of the weld region. The SEM image of the weld seam (Figure 5a) reveals a dendritic grain structure, with numerous precipitates distributed both along grain boundaries and within grains. SAED and EDS analyses (Figure 5b) indicate that the grain-boundary precipitates are Ti-rich MC carbides (Figure 5d), with relatively large phase sizes ranging from approximately 500 nm~1 μm. These precipitates are surrounded by chromium-rich M_23_C_6_ carbides (Figure 5e), with a size of approximately 100 nm. The TEM image of intragranular regions (Figure 5c) shows a high density of fine, particle-like precipitates that impede dislocation motion. The interaction between these precipitates and dislocations is believed to contribute to the increased microhardness observed in the weld metal.

#### 3.1.4. Microstructures at the Interface

No heterogeneous mixing zone or distinct transition layer was observed near the interface. Instead, significant co-crystallization occurred between the weld metal and the base metal, resulting in the formation of a dense solidification grain boundary in the interfacial region. Because both the weld and base metals are compositionally similar Ni-based face-centered cubic (FCC) austenitic high-temperature alloys, effective mutual mixing was achieved under the combined influence of arc forces and surface tension. Consequently, no obvious compositional or microstructural transition zone was detected at the interface. This observation further confirms the excellent compatibility between the weld metal and base metal during welding, consistent with previous studies on interfacial behavior in high-temperature alloy joints [28,29], and supports the interpretation presented in Section 3.1.

**Figure 6:**
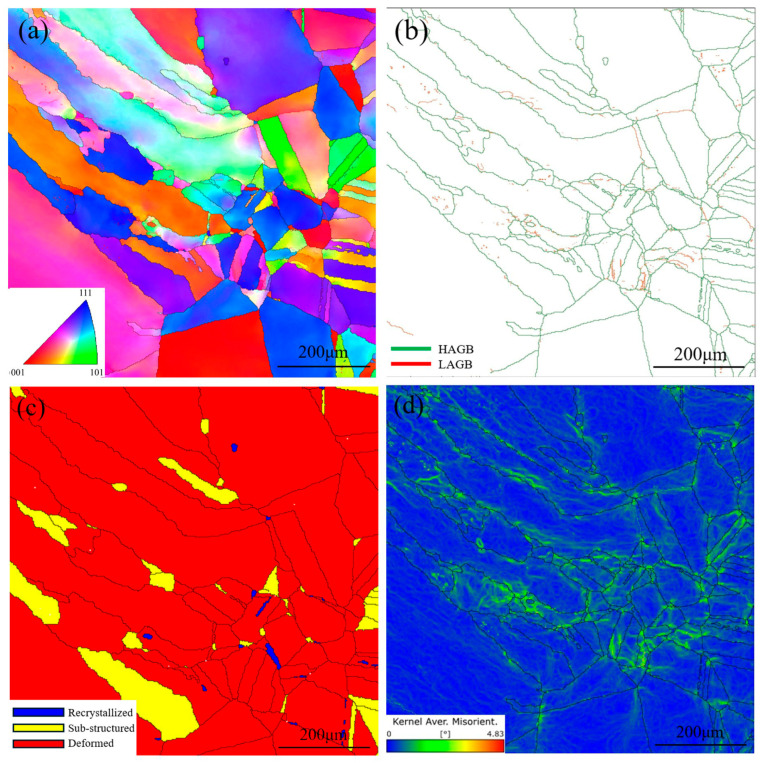
EBSD analysis results of the interface: (**a**) IPF analysis result of the interface; (**b**) grain boundary character of the interface; (**c**) grain type distribution of the interface; (**d**) KAM analysis result of the interface.

As shown in Figure 6, the low heat input of the gas tungsten arc welding (GTAW) process and the steep temperature gradient across the joint promote the formation of numerous columnar dendrites in the weld metal during solidification. These columnar dendrites exhibit pronounced competitive growth behavior, arising from nonuniform heat input during arc heating, which aligns the preferred dendrite growth direction with the maximum temperature gradient. When dendrites with differing orientations intersect, competitive growth occurs. Figure 6a shows that the base-metal side of the interface contains cellular crystals of varying sizes, whereas coarse columnar dendrites dominate the fusion zone. Figure 6b reveals the presence of numerous low-angle grain boundaries (LAGBs) in the base metal near the interface, primarily attributed to residual stresses generated during cooling. The LAGBs within the columnar dendrites on the weld side originate from elemental segregation during solidification.

Electron backscatter diffraction (EBSD) grain-orientation mapping (Figure 6c) indicates that most regions of the welded joint (red) consist of deformed grains characterized by high intragranular misorientation gradients and dislocation densities, suggesting incomplete recrystallization. A smaller fraction of substructured grains (yellow) and recrystallized grains (blue) appear mainly in areas of localized high strain, indicating the onset of dynamic recovery and recrystallization. Kernel average misorientation (KAM) analysis (Figure 6d) further reveals localized plastic-strain concentration on the base-metal side of the interface. This behavior arises from differences in the physical properties of the two materials. During weld solidification, the fusion zone near the base-metal side solidifies first. As solidification proceeds, shrinkage-induced stresses develop within the weld metal. When these stresses exceed the local yield strength, plastic deformation occurs at the interface. Because the stress is insufficient to deform the base metal and the weld center remains at elevated temperature, the deformation is primarily confined to the interfacial region.

### 3.2. Mechanical Properties

#### 3.2.1. Microhardness

**Figure 7:**
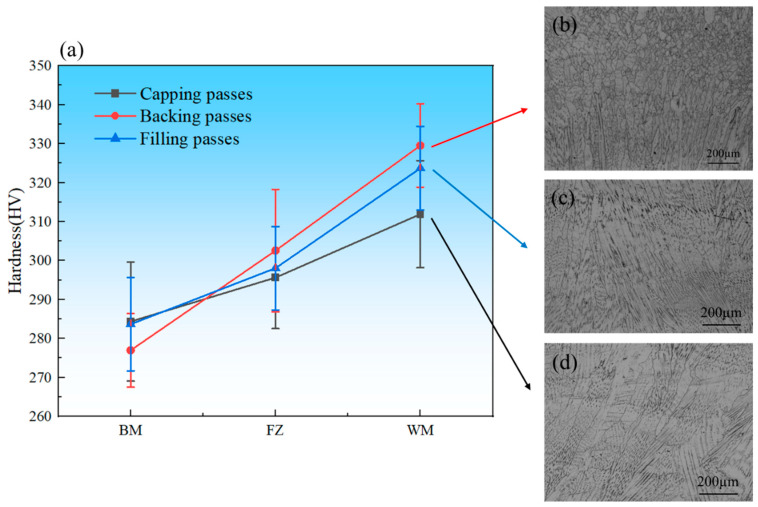
(**a**) Microhardness distribution of the welded joint; (**b**) Metallographic structure of the backing passes; (**c**) Metallographic structure of the filling passes; (**d**) Metallographic structure of the capping passes.

Microhardness measurements were performed across different regions of the welded joint, and the results are shown in Figure 7. At each microhardness sampling position, at least five indentations were made with a load of 200 g and a dwell time of 5 s. The error bars in Figure 7 indicate the standard deviations of the measurements at each location. The microhardness at 5~7 mm from the fusion line on the cap weld side is representative of the overall microhardness of the base material (see Figure 7). This sampling strategy avoids local hardness differences (311–329 HV) between different welds, ensuring that the measured tensile properties of the joint reflect the overall properties of the welded plate, rather than local inhomogeneities. Within the weld seam, the backing pass exhibited the highest hardness (approximately 329 HV), followed by the fill pass (approximately 323 HV) and the cap pass (approximately 311 HV). As the first welding layer, the backing pass cooled rapidly, resulting in higher hardness. In contrast, the cap pass cooled more slowly and experienced multiple thermal cycles, leading to microstructural tempering and softening, which markedly reduced hardness. Previous studies [30,31] have shown that variations in microhardness are closely associated with grain-refinement mechanisms. Compared with single-pass welding, multi-pass welding promotes grain refinement due to several factors: (a) subsequent thermal cycles induce partial recrystallization and refinement of grains formed in previous passes; (b) the overall heat input gradually decreases as welding progresses, thereby suppressing excessive grain growth; and (c) the preceding passes create a preheating effect that prolongs the t_8/5_ time (the cooling time from 1073 K to 773 K), influencing microstructural evolution. As shown in Figure 7b, the rapid solidification of the weld seam produces fine columnar or equiaxed grains, resulting in significantly higher hardness compared with the base metal and HAZ—a trend consistent with the Hall–Petch relationship. Furthermore, the dislocation–precipitate interactions discussed in Section 3.1.2 also contribute to the elevated hardness of the weld seam. According to literature reports [32], the transition zone exhibits marked microstructural and property changes caused by welding heat input. Its hardness is generally lower than that of the weld metal but slightly higher than or comparable to the base metal. As shown in Figure 7c, variations among the different weld passes influence both the extent and hardness response of the transition zone. Local overheating leads to grain coarsening, thereby reducing hardness [33]. The base metal, characterized by its stable microstructure, is unaffected by welding thermal cycles and thus undergoes little grain refinement, as illustrated in Figure 7d. The precipitation accumulation and stress concentration discussed in Section 3.1.1—which promote crack initiation—reasonably explain the lower microhardness observed in this region.

#### 3.2.2. Tensile Properties

**Figure 8:**
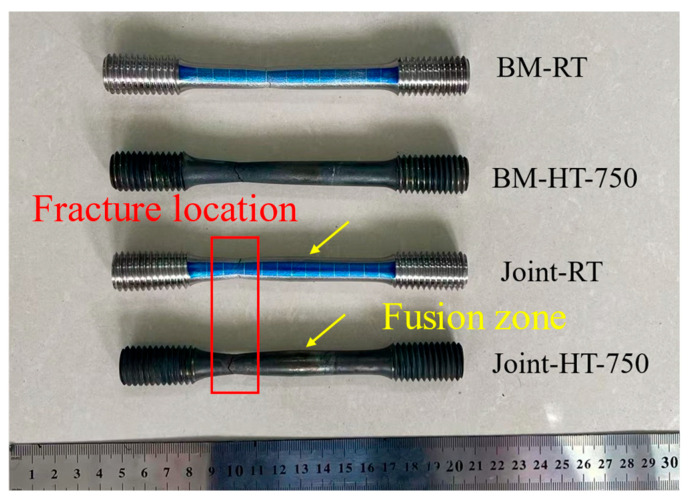
Fracture location of the specimens.

Table 2 summarizes the tensile properties of the stabilized welded joints and base metal, including yield strength (YS), ultimate tensile strength (UTS), total elongation (EL), and reduction in area (AR). For clarity, the room-temperature tensile tests for the base metal and welded joint are denoted as BM-RT and Joint-RT, respectively, while the high-temperature tensile tests at 750 °C are referred to as BM-HT-750 and Joint-HT-750. The elevated temperature of 750 °C was selected because it is representative of the design operating temperature range of Inconel 617 components in advanced high-temperature reactor systems and high-temperature heat exchangers, as specified in ASME Section III [15], Division 5 design guidelines. Analysis of the tensile test results showed that the tensile strengths of the welded joint at room temperature and at high temperature were 920 MPa and 605.5 MPa, respectively, with corresponding joint efficiencies of 117% and 121%. As shown in Figure 8, one specimen was selected for each experimental condition for subsequent deformation mechanism analysis. The fracture location under both test conditions was located within the base material. This indicates that the strength of the base material is lower than that of the welded joint, consistent with the trend of the microhardness test results. Compared with room temperature, the yield strength and tensile strength of both the base material and the welded joint decreased at 750 °C, mainly due to recovery and softening phenomena, as well as increased damage sensitivity at high temperatures [10]. The joint efficiencies of 117% at room temperature and 121% at 750 °C indicate a clearly overmatched joint, where the weld metal is stronger than the base metal. Similar or even higher joint efficiencies have been reported for Inconel 617 and related Ni–Cr–Co-based weldments produced by optimized GTAW or CMT processes, in which grain refinement and a high density of carbides in the weld metal provide additional strengthening compared with the base plate [10,25,26,27,32]. From a structural design perspective, such overmatching is generally desirable because it shifts plastic deformation and final fracture away from the weld metal to the base metal, thereby avoiding premature weld failure. However, under long-term high-temperature or cyclic loading, this strength mismatch may also lead to strain localization in the softer base metal or HAZ, and a detailed assessment of creep and fatigue damage accumulation in these regions is therefore required for component qualification in nuclear applications. The hardness gradient across the welded joint (Figure 7), with higher hardness in the weld metal and slightly lower hardness in the base metal, suggests that under service conditions involving cyclic or thermomechanical loading, plastic strain will preferentially localize in the softer base metal and any adjacent HAZ rather than in the weld metal. This behavior is consistent with the observed fracture locations and is beneficial in terms of avoiding weld metal failure. Nevertheless, in long-term operation of nuclear components, the combined effects of strength overmatching and hardness gradients on cyclic softening, ratcheting, and creep–fatigue damage evolution should be carefully evaluated in future life assessment studies.

### 3.3. Fracture Morphology and Deformation Mechanism Analysis of Tensile Specimens

The fracture morphology of the tensile specimens reveals the fracture modes and characteristic deformation behavior of the welded joint under different conditions. In this study, fractures in both the base metal and welded-joint specimens occurred within the base metal. Because the testing conditions were identical for both specimen types, no significant differences in fracture morphology or fracture mode were observed. Therefore, the following discussion focuses on the macroscopic fracture morphology of the welded-joint tensile specimens.

#### 3.3.1. Fracture Morphology

**Figure 9:**
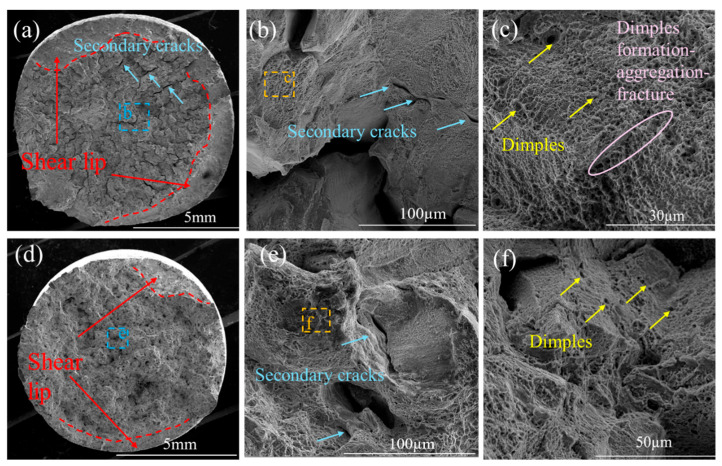
(**a**–**c**) Fracture surface micrographs of Joint-RT after tensile testing; (**d**–**f**) Fracture surface micrographs of Joint-HT-750 after tensile testing.

As shown in Figure 9a, the macroscopic fracture surface at room temperature exhibits a typical cup-and-cone morphology, consisting of a central fibrous region surrounded by a shear-lip region. At high temperature (Figure 9d), the fracture surface displays a similar fibrous–shear-lip configuration but with a noticeably coarser appearance, indicating that fracture occurred through progressive crack propagation rather than an instantaneous event. Compared with the room-temperature specimen, the shear-lip region is significantly reduced, and the fracture surface features more pronounced ductile dimples. Ductile fracture generally manifests as a cup-and-cone surface, arising from localized plastic deformation after the material reaches its ultimate tensile stress. Initially, strain concentrates in a specific region, causing gradual reduction in the cross-sectional area and the formation of necking. With continued deformation, fracture propagates along the cup-and-cone profile, completing the failure process. The central fibrous region contains evident secondary cracks, while a relatively narrow shear-lip region surrounds it. As shown in Figure 9b, the fibrous region exhibits ductile intergranular fracture with secondary cracks distributed along grain boundaries. Irregular depressions and tearing ridges indicate substantial plastic flow and localized deformation during fracture, consistent with ductile behavior. In Figure 9c, numerous closely spaced dimples characteristic of the microvoid-coalescence mechanism are observed, suggesting that microvoids nucleated, grew, and eventually coalesced during tensile loading, leading to final rupture. At high temperature (Figure 9f), the dimples are predominantly spherical, deep, and densely packed, implying that the material absorbed considerable plastic deformation energy prior to fracture. The tensile load direction is approximately perpendicular to the fracture surface, further confirming the ductile-tensile fracture mode. Moreover, the absence of cleavage facets and river-pattern striations rules out the possibility of a cleavage-type fracture.

#### 3.3.2. Deformation Microstructure and Precipitation Analysis of the Joint

**Figure 10:**
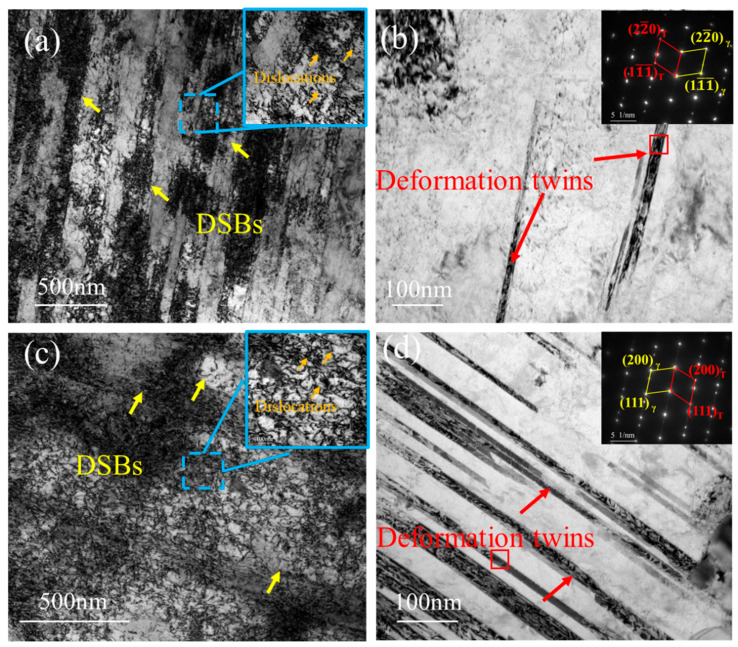
(**a**) TEM image of slip bands and dislocations during tensile deformation of Joint-RT; (**b**) TEM image of deformation twins in Joint-RT; (**c**) TEM image of slip bands and dislocations during tensile deformation of Joint-HT-750; (**d**) TEM image of deformation twins in Joint-HT-750.

The deformation microstructure developed during tensile testing is critical for elucidating the fracture mechanism. Therefore, the post-deformation microstructures of the specimens were thoroughly characterized. As shown in Figure 10a, after room-temperature deformation, the microstructure is dominated by parallel dislocation slip bands (DSBs), with a small number of deformation twins identified by SAED (Figure 10b). After high-temperature deformation, a limited number of parallel DSBs remain visible (Figure 10c). In addition, pronounced dislocation entanglement is observed between slip bands, accompanied by numerous deformation twins (Figure 10d).

For FCC alloys such as Inconel 617, a relatively low stacking-fault energy (SFE) in combination with high resolved shear stresses favors the activation of deformation twinning as a complementary mechanism to slip. When dislocation motion is strongly impeded by dislocation pile-ups, unfavorable slip orientations, or the pinning effect of carbides, the local shear stress can exceed the critical value for twin nucleation. In the present work, all tests were conducted at a constant strain rate ($\dot{\varepsilon }$ = 1.0 × 10^−3^ s^−1^), so the observed transition from slip-dominated deformation at room temperature to twinning-dominated deformation at 750 °C should be interpreted as the combined result of temperature, strain rate, and precipitate–dislocation interactions rather than SFE alone [34]. At higher strain rates, slip would be expected to remain more active, whereas at lower strain rates and higher temperatures, diffusion-assisted processes and dynamic recovery may further facilitate twin formation and growth.

At room temperature, the dominant deformation mechanism is dislocation slip, with limited twinning activity. Because Inconel 617 possesses relatively high SFE at room temperature, dislocation glide is favored over twinning. Twinning requires higher shear stress, and it occurs locally when slip is obstructed and local stress concentrations develop. By contrast, at high temperature (Figure 10c), deformation twinning becomes the primary deformation mode, accompanied by minor slip activity. The SFE of Inconel 617 decreases with increasing temperature, leading to dislocation dissociation into partials that readily form twins [35,36]. Under such conditions, dislocations can also climb more easily, preventing their accumulation and the formation of pronounced slip bands. Furthermore, during high-temperature deformation, dynamic recovery and even dynamic recrystallization may occur, reducing the dislocation density and suppressing the observable development of slip bands.

**Figure 11:**
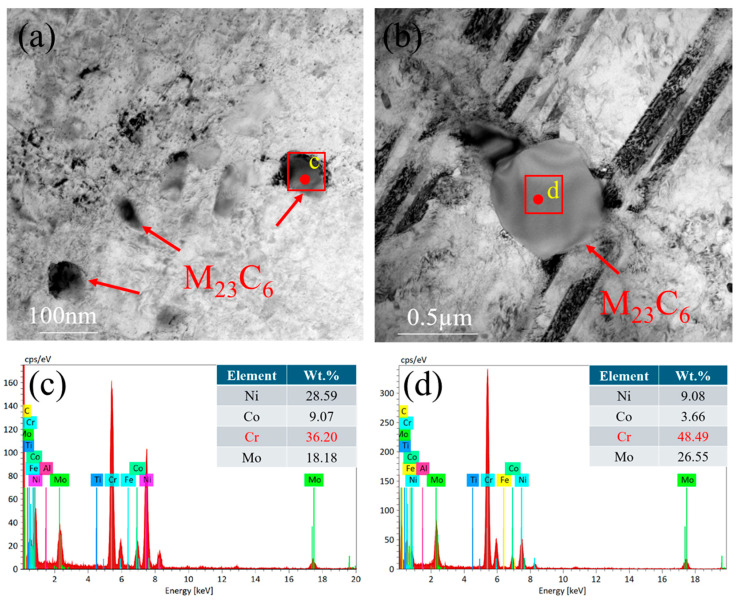
(**a**) TEM image of Cr-rich precipitates during tensile deformation of Joint-RT; (**b**) TEM image of Cr-rich precipitates during tensile deformation of Joint-HT-750. (**c**) EDS of M_23_C_6_ precipitates; (**d**) EDS of M_23_C_6_ precipitates.

To further investigate the evolution of precipitates under tensile deformation, TEM was employed, complemented by EDS for compositional characterization. Figure 11a shows the precipitates in the joint after room-temperature deformation, where intergranular carbides are identified by EDS as Cr-rich M_23_C_6_ (Figure 11c). After high-temperature deformation (Figure 11b), precipitates are observed along twin boundaries and are likewise confirmed by EDS to be Cr-rich M_23_C_6_ (Figure 11d). These results indicate that M_23_C_6_ remains the dominant precipitate phase after tensile deformation. No Mo-rich M_6_C or Ti-rich MC carbides were detected in the TEM analysis. This predominance of M_23_C_6_ is consistent with previous reports on Inconel 617 [37,38,39].

Studies of long-term high-temperature exposure (649–1093 °C) have shown that M_23_C_6_ is the principal stable phase in Inconel 617, with negligible formation of MC or M_6_C [40,41]. Within the 600–950 °C range, Mo atoms exhibit a solubility limit of approximately 10–15%, whereas Cr atoms show a significantly higher limit of 32–43%. Because of the much lower solubility of Mo, pre-existing carbides are unlikely to dissolve into the matrix and reprecipitate as new carbides. Moreover, at elevated temperatures, the M_23_C_6_ precipitates located between twin boundaries serve as obstacles to dislocation glide, altering dislocation trajectories and restricting slip. This obstruction likely promotes deformation twinning within the grains under high-temperature conditions.

#### 3.3.3. Overview of the Deformation Mechanisms in the Tensile Behavior of the Joint

**Figure 12:**
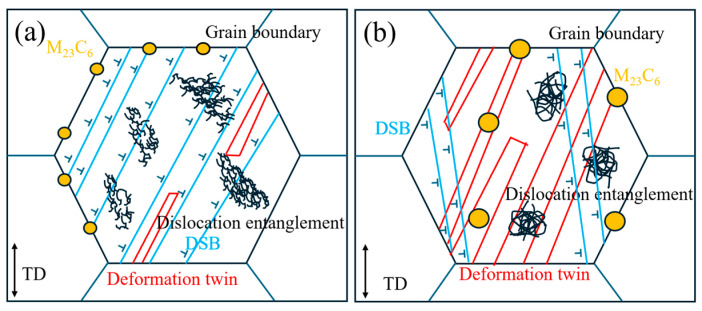
Schematic of the tensile deformation mechanisms in Inconel 617 welded joints. (**a**) Room tempertaure; (**b**) 750 °C.

In summary, the deformation mechanisms of the Inconel 617 welded joint after tensile testing at room and elevated temperatures are illustrated in Figure 12. At room temperature, deformation is dominated by planar slip and dislocation entanglement. As shown in Figure 12a, numerous parallel slip bands develop within the grains, accompanied by pronounced dislocation tangling between these bands. With increasing strain, dislocation motion becomes progressively constrained, leading to the formation of deformation twins. These twins facilitate plastic deformation by redistributing local stresses and alleviating stress concentrations. Moreover, interactions between M_23_C_6_ precipitates and the matrix act as obstacles to dislocation glide, increasing the resistance to plastic flow and influencing the overall tensile response.

At elevated temperatures, the deformation behavior changes markedly, as depicted in Figure 12b. The reduced stacking-fault energy promotes deformation twinning, while the higher mobility of dislocations leads to fewer slip bands. Consequently, deformation twinning becomes the dominant mechanism under high-temperature conditions, accompanied by limited slip activity. Overall, plastic deformation at room temperature is primarily governed by dislocation slip and entanglement, with minor twinning, whereas at elevated temperature, deformation twinning predominates. Additionally, M_23_C_6_ precipitates serve as pinning sites for dislocations, further modifying their motion paths and contributing to the observed deformation behavior. The above observations establish a direct link between the deformation mechanisms and the macroscopic tensile response summarized in Table 2. At room temperature, planar slip combined with strong dislocation–precipitate interactions provides high yield and tensile strengths, while the limited activation of deformation twinning and the homogeneous distribution of slip bands allow relatively high elongation. At 750 °C, the transition to twinning-dominated deformation, assisted by dynamic recovery, leads to a moderate reduction in strength but maintains substantial ductility through sustained work hardening. The fine dendritic microstructure and dense carbide distribution in the weld metal contribute to the overmatching joint efficiencies observed for the welded joints in both temperature regimes. It should be noted that the present work investigates tensile behavior at a single elevated temperature (750 °C). At higher temperatures approaching 850–950 °C, diffusion-controlled processes such as dynamic recovery, dynamic recrystallization, and coarsening or redistribution of carbides are expected to become more pronounced, which may further modify the balance between slip and twinning and reduce work-hardening capacity. A systematic temperature-dependent study, including tensile and creep testing at multiple temperatures, is therefore required to fully map the deformation mechanisms of GTAW-welded Inconel 617 joints over the entire service range and will be the subject of future work.

## 4. Conclusions

The microstructure of the Inconel 617 alloy and ERNiCrCoMo-1 filler wire GTAW welded joint was studied. The microhardness and uniaxial tensile properties of the joint at room temperature and 750 °C were systematically tested, and the tensile fracture was analyzed. A limitation of the present study is that only monotonic tensile loading was considered, whereas components in nuclear and high-temperature energy systems are predominantly subjected to long-term creep or creep–fatigue interaction. Although the microstructural features identified here—such as the fine dendritic substructure and dense carbide distribution in the weld metal—are expected to influence creep strength and damage evolution, no direct creep data are currently available for the investigated GTAW joints. Future work will therefore focus on systematic creep and creep–fatigue testing of Inconel 617 welded joints over a range of temperatures and stresses, combined with in-depth microstructural analysis of damage development in the weld metal, HAZ, and base metal. Based on the research findings, the following conclusions can be drawn:

(1) The welded joint shows excellent metallurgical compatibility, with co-crystallization at the interface and no observable transition zone. M_23_C_6_ and MC carbides formed during thermal cycling near the fusion line pin dislocations, which enhances strength while slightly reducing plasticity. Plastic deformation mainly occurs in the base metal, indicating strong interfacial bonding and structural stability.
(2) The welded joints exhibit excellent mechanical integrity, achieving tensile strengths of 920 MPa at room temperature and 605.5 MPa at 750 °C, corresponding to joint efficiencies of 117% and 121%, respectively. All fractures occur within the base metal, confirming the high quality and reliability of the welds.
(3) The hardness of the welded joint varies significantly across different welding passes, with the highest value of 329 HV observed in the backing pass and the lowest of 311 HV in the cap pass. The weld metal exhibits higher hardness than the base metal, primarily due to grain refinement and precipitation strengthening during welding. The elevated hardness and strength of the weld region are attributed to grain refinement, precipitation hardening, and dislocation–precipitate interactions, consistent with the Hall–Petch relationship. The dense distribution of M_23_C_6_ precipitates and the fine dendritic substructure collectively contribute to local strengthening.
(4) Deformation behavior is strongly temperature-dependent. At room temperature, plasticity is governed by planar slip and dislocation entanglement, with limited twinning. At elevated temperatures, deformation twinning becomes the dominant mechanism due to the reduced stacking-fault energy and the pinning effect of M_23_C_6_ carbides on dislocation motion. These mechanisms collectively account for the excellent high-temperature strength and ductility of the GTAW-welded Inconel 617 joints.
(5) From a technological standpoint, the combination of a low-heat-input GTAW procedure and compositionally compatible ERNiCrCoMo-1 filler produces overmatched Inconel 617 welded joints with a very narrow HAZ and a refined weld metal microstructure. These features are desirable for high-temperature nuclear components, as they reduce the likelihood of weld metal failure and offer a favorable basis for subsequent creep and creep–fatigue life assessment.

## Figures and Tables

**Table 1:** Chemical composition (wt.%) of BM and WM.

	C	Si	Cr	Mo	Ti	Al	Co	Fe	Ni
BM	0.065	0.021	21.81	8.73	0.41	1.10	12.03	0.19	Bal.
WM	0.05	0.1	21.5	9.0	0.3	1.3	11.0	0.5	Bal.

**Table 2:** Tensile results for the Joint and BM.

Sample No.	Tensile Properties			
	UTS (MPa)	YS (MPa)	EL (%)	AR (%)
BM-RT	870 ± 5	392 ± 4	48 ± 1	51 ± 2
BM-HT-750	560 ± 15	301 ± 25	47.5 ± 2	45.5 ± 3
Joint-RT	920 ± 5	459 ± 10	37 ± 1	51 ± 2
Joint-HT-750	605.5 ± 6	364 ± 15	30 ± 4	43.5 ± 4

UTS: Ultimate tensile strength, YS: Yield strength, EL: Elongation, AR: Area reduction.

## Data Availability

The original contributions presented in this study are included in the article. Further inquiries can be directed to the corresponding authors.
